# Restoration of Motility in Acrosome‐Reacted Boar Spermatozoa Induced by the Ionophore 4‐Br‐A23187

**DOI:** 10.1111/rda.70236

**Published:** 2026-09-30

**Authors:** David Martín‐Hidalgo, Violeta Calle‐Guisado, Beatriz Macías‐García, Lauro González‐Fernández

**Affiliations:** ^1^ SINTREP Group, Departamento de Fisiología, Facultad de Medicina y Ciencias de la Salud Universidad de Extremadura Badajoz Spain; ^2^ BD Group, Departamento de Anatomía, Biología Celular y Zoología, Facultad de Medicina y Ciencias de la Salud Universidad de Extremadura Badajoz Spain; ^3^ MINVET Group, Departamento de Medicina Animal, Facultad de Veterinaria Universidad de Extremadura Cáceres Spain; ^4^ SINTREP Group, Departamento de Bioquímica y Biología Molecular y Genética, Facultad de Veterinaria Universidad de Extremadura Cáceres Spain

**Keywords:** acrosome reaction, boar, motility, sperm

## Abstract

The acrosome reaction (AR) is a calcium‐dependent exocytotic event required for successful fertilization. Calcium ionophores are commonly used to induce AR in vitro; however, this treatment is associated with a significant loss of sperm motility. The aim of this study was to evaluate the recovery of motility in boar spermatozoa after ionophore‐induced AR and to assess the role of extracellular calcium. Boar spermatozoa were incubated with 10 μM 4‐Br‐A23187 to induce AR. Then, spermatozoa were washed and incubated for 30 min in a recovery medium containing either 1 mM calcium or 5 mM EGTA. The AR was analysed by flow cytometry, and motility parameters were assessed using a CASA system. Ionophore treatment induced AR and caused a marked reduction in sperm motility, which was partially recovered in the presence of 5 mM EGTA, but not in the presence of 1 mM calcium. These results suggest that extracellular calcium inhibits the restoration of motility after ionophore‐induced AR in boar spermatozoa.

## 1 Introduction

The acrosome reaction (AR) is a calcium‐dependent exocytic process that plays a crucial role during fertilization. This event allows the sperm plasmalemma to fuse with the oocyte membrane, being therefore essential for successful fertilization. Under in vivo conditions, the AR occurs after sperm capacitation and induction of hyperactivated motility within the female reproductive tract and is finely regulated by extracellular calcium levels and intracellular signalling pathways (Aldana et al. 2021). The AR can be artificially induced in vitro by different stimuli, including calcium ionophores. These compounds increase intracellular calcium concentrations by facilitating calcium influx across the plasma membrane, thereby triggering acrosomal exocytosis (Mata‐Martinez et al. 2021). In boar spermatozoa, previous studies have described that incubation with the calcium ionophore 4‐Br‐A23187 induces the AR (Martin‐Hidalgo et al. 2024).

During natural fertilization, in some species such as mice, the acrosome may be lost while spermatozoa progress through the oviductal ampulla being still able to reach the oocyte (La Spina et al. 2016). However, induction of the AR by ionophores causes a complete loss of sperm motility in several species, including mouse (Tateno et al. 2013) and bovine (Osycka‐Salut et al. 2024). Interestingly, the motility can be recovered after removal of the ionophore and incubation in a capacitating medium containing calcium, BSA, and bicarbonate. Under these conditions, mouse and bovine spermatozoa recover motility and acquire a hyperactivated pattern (Osycka‐Salut et al. 2024; Tateno et al. 2013).

The recovery of motility in acrosome‐reacted spermatozoa represents a valuable experimental model to study the motility patterns of acrosome‐reacted sperm in vitro. Therefore, the objective of this study was to analyse the recovery of motility in boar spermatozoa after ionophore‐induced AR and to evaluate the effect of the presence of calcium in the recovery medium on sperm motility.

## 2 Materials and Methods

### 2.1 Sperm Processing

Seminal doses from Duroc boars were obtained from a commercial boar station (Tecnogenext S.L., Mérida, Spain). A total of six independent experiments (*n* = 6) were performed using seminal doses from six different boars. Seminal doses were centrifuged at 900 × *g* for 4 min, washed with PBS, and diluted in Tyrode's capacitating medium (TCM) to achieve a final concentration of approximately 50 × 10^6^ spermatozoa/ml (500 μL final volume). TCM was prepared with the following composition: 96 mM NaCl, 4.7 mM KCl, 0.4 mM MgSO_4_, 0.3 mM NaH_2_PO_4_, 5.5 mM glucose, 1 mM sodium pyruvate, 21.6 mM sodium lactate, 20 mM HEPES, 15 mM NaHCO_3_, 1 mM CaCl_2_ and 3 mg/mL BSA, and the pH was adjusted to 7.25. Spermatozoa were first incubated for 5 min at 38.5°C in a water bath under atmospheric air, followed by a 20‐min incubation with 10 μM 4‐Br‐A23187 to induce the AR (Martin‐Hidalgo et al. 2024). The control group was incubated under the same experimental conditions as treated samples but without 4‐Br‐A23187 and with the corresponding vehicle concentration (0.2% DMSO). After incubation, spermatozoa were centrifuged at 2500 × *g* for 1 min, washed with 1 mL of the recovery medium (TCM medium without pyruvate, lactate and BSA) supplemented with 1 mg/mL PVA and either 5 mM EGTA or 1 mM calcium, depending on the treatment. After the washing step with recovery medium (EGTA or calcium), the samples were centrifuged again and resuspended in 450 μL of recovery medium (EGTA or calcium) and incubated for 30 min at 38.5°C before assessment.

### 2.2 Flow Cytometry Analysis

The AR was evaluated by flow cytometry using an ACEA Novocyte flow cytometer (ACEA Biosciences Inc., San Diego, CA, USA). Peanut agglutinin conjugated with fluorescein isothiocyanate (PNA‐FITC) was used in combination with propidium iodide (PI), as previously reported (Martin‐Hidalgo et al. 2022). The AR was expressed as the percentage of live acrosome‐reacted spermatozoa (PNA^+^/PI^−^).

### 2.3 Motility Evaluation

Sperm motility was assessed using a CASA system (ISAS 1.0.6; Proiser S.L., Valencia, Spain). At least three microscopic fields and a minimum of 300 spermatozoa per sample were evaluated. The parameters evaluated included total motility (TM; %), curvilinear velocity (VCL; μm/s), amplitude of lateral head displacement (ALH; μm), and linearity (LIN; %).

### 2.4 Statistical Analysis

Statistical analyses were performed using SigmaPlot ver. 12.0 for Windows (Systat Software, Chicago, IL, USA). Data normality and homogeneity of variances were assessed using the Shapiro–Wilk and Levene's tests, respectively. Differences among groups were evaluated by one‐way ANOVA followed by Fisher's LSD *post hoc* test. Comparisons between two groups were performed using Student's *t*‐test or the Mann–Whitney *U* test depending on data distribution. Statistical significance was set at *p* < 0.05.

## 3 Results

Our results showed that treatment with 4‐Br‐A23187 induced the AR in approximately 65% of spermatozoa, whereas approximately 8% remained viable with an intact acrosome (Figure 1A,B). This treatment resulted in a complete loss of motility (Figure 1C), in agreement with our previous findings (Martin‐Hidalgo et al. 2024). After the recovery period, boar spermatozoa incubated in the presence of 1 mM calcium remained immotile, whereas those incubated with 5 mM EGTA partially recovered their motility, reaching approximately 50% total motility (Figure 2A). This recovery was accompanied by a significant increase in ALH and a decrease in VCL and LIN (Figure 2B).

**FIGURE 1:**
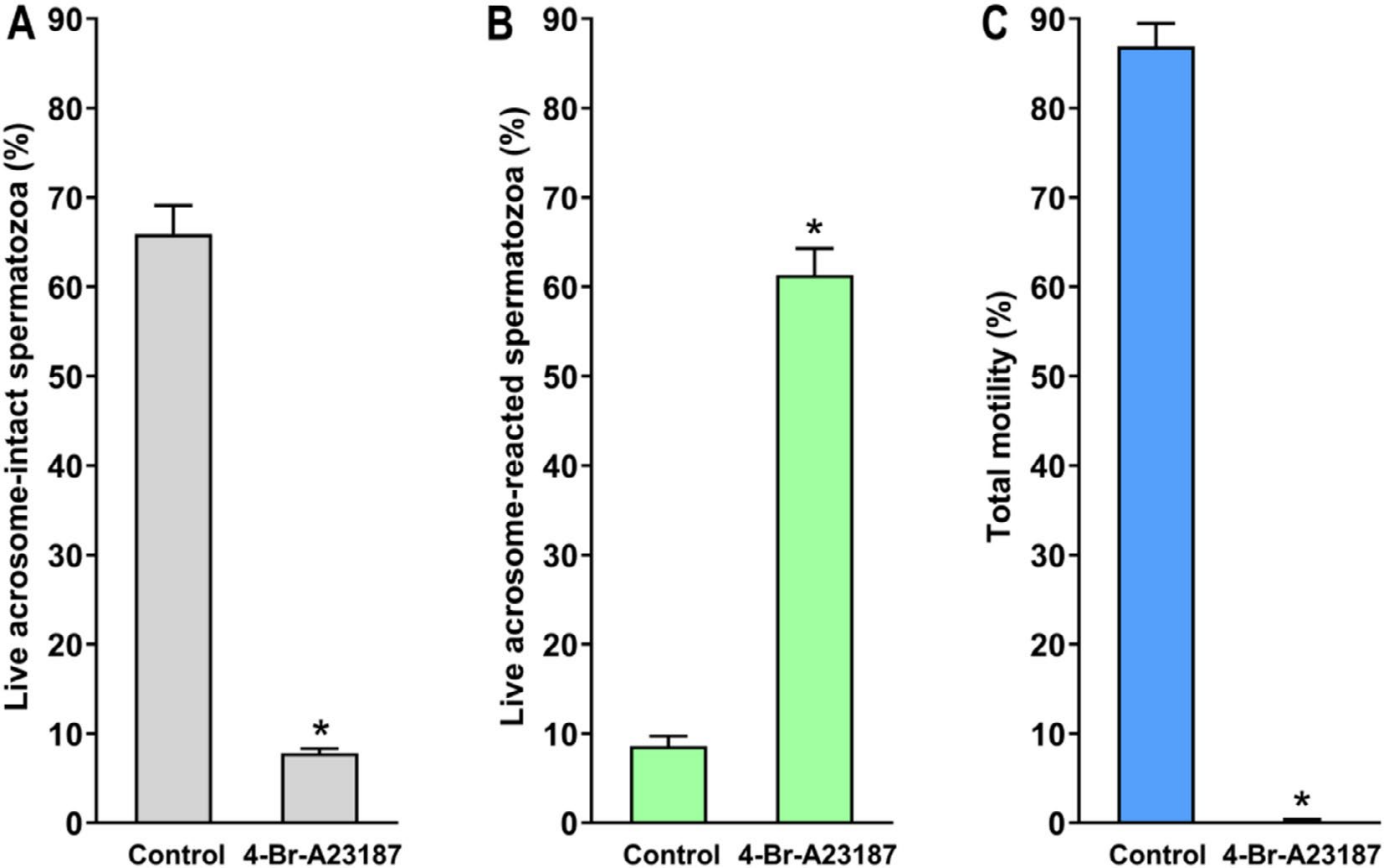
Effects of 4‐Br‐A23187 on acrosome status and total motility in boar spermatozoa. Spermatozoa were incubated in TCM for 20 min at 38.5°C in the presence or absence of 10 μM 4‐Br‐A23187. (A) Percentage of live acrosome‐intact spermatozoa. (B) Percentage of live acrosome‐reacted spermatozoa. (C) Percentage of total motility. Data are expressed as mean ± standard error of the mean (SEM). (*) Indicates statistically significant differences (*p* < 0.05; *n* = 6).

**FIGURE 2:**
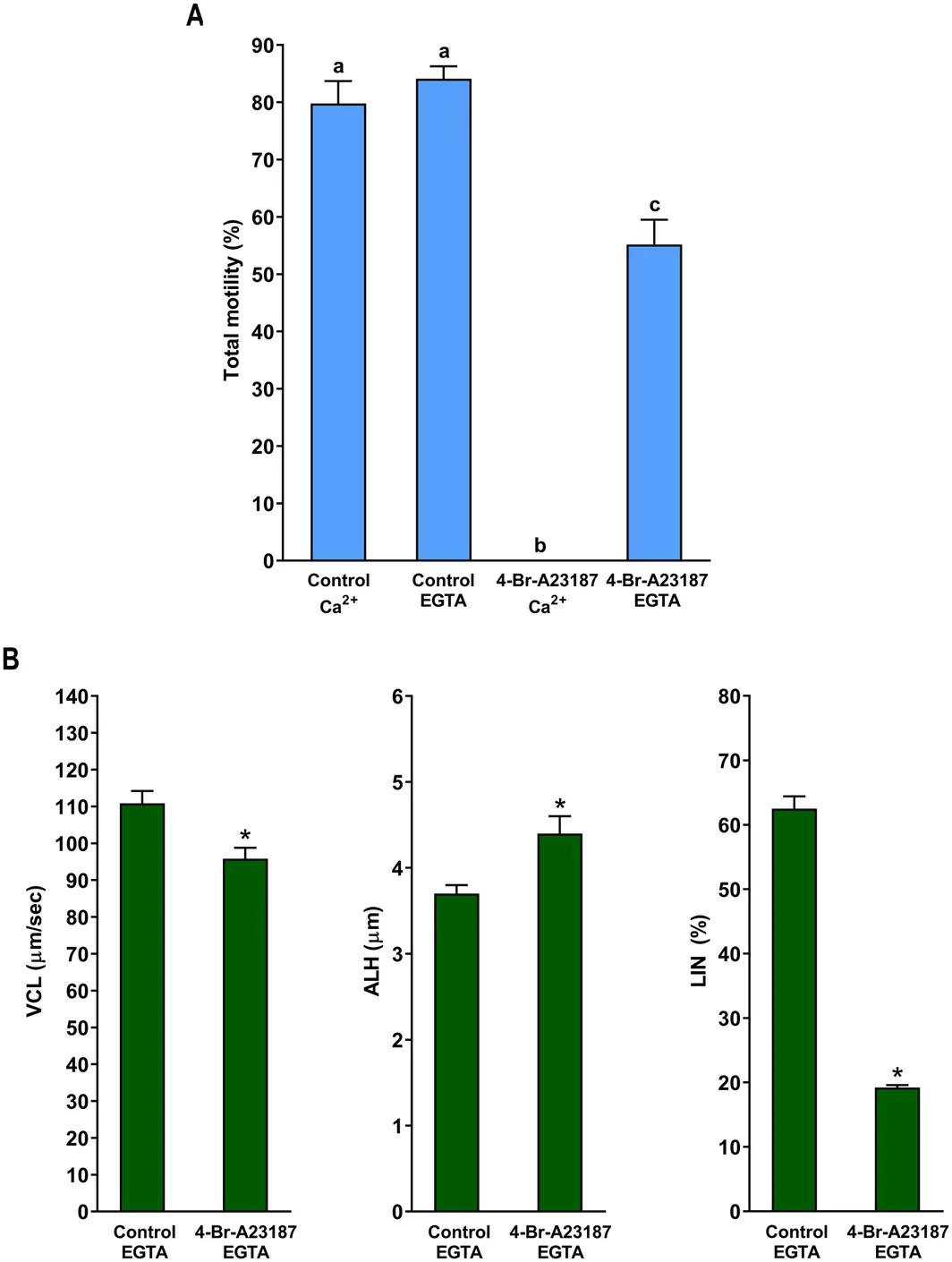
Effects of calcium or EGTA on motility parameters of boar spermatozoa in recovery medium following 4‐Br‐A23187 treatment. Following induction of the acrosome reaction, spermatozoa were incubated for 30 min in the presence of 1 mM calcium or 5 mM EGTA. (A) Percentage of total motility. Different letters indicate statistically significant differences among groups (*p* < 0.05; *n* = 6). (B) Motility parameters of boar spermatozoa incubated in the presence of 5 mM EGTA. Values of VCL (μm/s), ALH (μm), and LIN (%). Data are presented as mean ± SEM. (*) Indicates statistically significant differences (*p* < 0.05; *n* = 6).

## 4 Discussion

Our study demonstrates that motility can be partially restored after ionophore treatment following incubation in a medium containing EGTA. To the best of our knowledge, this is the first report describing this effect in boar spermatozoa. Notably, after ionophore treatment, only a small proportion of spermatozoa (approximately 8%) remained viable with an intact acrosome (PNA^−^/PI^−^). Therefore, the observed recovery of motility (approximately 50%) is most likely attributable mainly to live acrosome‐reacted spermatozoa (PNA^+^/PI^−^). Previous studies in mouse and bovine spermatozoa have reported that immotile spermatozoa following ionophore treatment are able to recover motility under capacitating conditions in the presence of calcium (Tateno et al. 2013; Osycka‐Salut et al. 2024). In contrast, under our experimental conditions, boar spermatozoa remained immotile after ionophore removal and resuspension in a calcium‐containing medium. Interestingly, motility recovery was only observed in the presence of 5 mM EGTA, indicating that extracellular calcium prevents the restoration of motility following ionophore exposure. This discrepancy may be related to a limited capacity of calcium extrusion mechanisms in boar spermatozoa to reduce intracellular calcium levels after treatment with 4‐Br‐A23187. In this context, plasma membrane Ca^2+^‐ATPase (PMCA) has been described in spermatozoa (Aldana et al. 2021), and it is possible that boar spermatozoa contain a reduced number of PMCA pumps or lower pump activity compared to mouse spermatozoa. Consequently, at 1 mM extracellular calcium, these pumps may be unable to efficiently extrude calcium, resulting in impaired sperm motility. In contrast, the presence of extracellular EGTA may facilitate calcium efflux by reducing extracellular calcium availability and maintaining a gradient for calcium efflux, thereby contributing to the recovery of sperm motility in boar spermatozoa. In addition, intracellular calcium stores and internal calcium pumps, such as sarco/endoplasmic reticulum Ca^2+^‐ATPase (SERCA) (Aldana et al. 2021), have been described in spermatozoa and may also participate in the regulation of calcium homeostasis, contributing to the observed recovery of motility in the presence of EGTA. However, the precise mechanisms underlying this process remain unclear and require further investigation.

The recovered motility pattern partially resembles hyperactivated motility, which is typically characterized by increased VCL and ALH together with decreased LIN (Martin‐Hidalgo et al. 2018). However, in our experimental conditions, no increase in VCL was observed. Given that elevated VCL is a hallmark of hyperactivation, these results suggest that the recovered motility pattern may represent a different type of sperm movement rather than classical hyperactivation. Alternatively, these findings could be explained by increased adhesiveness of acrosome‐reacted spermatozoa compared to acrosome‐intact sperm, which may mechanically constrain their movement and prevent an increase in VCL.

## 5 Conclusions

In conclusion, our results demonstrate that the motility of acrosome‐reacted boar spermatozoa induced by 4‐Br‐A23187 can be restored after incubation in an EGTA‐containing medium for 30 min. These findings provide a potential experimental model to investigate motility in acrosome‐reacted boar spermatozoa. Further studies are required to elucidate the cellular and molecular mechanisms underlying this regulation.

## Author Contributions

Conceptualization, L.G.‐F.; methodology, L.G.‐F.; formal analysis, D.M.‐H. and L.G.‐F.; investigation, D.M.‐H. and L.G.‐F.; resources, B.M.‐G. and L.G.‐F.; writing – original draft preparation, D.M.‐H., V.C.‐G., B.M.‐G. and L.G.‐F.; writing – review and editing, D.M.‐H., V.C.‐G., B.M.‐G. and L.G.‐F.; supervision, L.G.‐F.; project administration, L.G.‐F.; funding acquisition, B.M.‐G. and L.G.‐F. All authors have read and agreed to the published version of the manuscript and to the order of authors.

## Funding

This work has been co‐funded by the European Union, European Regional Development Fund (85%), and Junta de Extremadura. Managing authority: Ministerio de Hacienda (Spain). Grant GR24163.

## Conflicts of Interest

The authors declare no conflicts of interest.

## Data Availability

The data that support the findings of this study are available from the corresponding author upon reasonable request.
